# Orbital clock concept: A novel anatomical zoning system for endoscopic vs. external orbital approaches

**DOI:** 10.1007/s00405-026-10155-6

**Published:** 2026-04-29

**Authors:** Mostafa Tarek Fayed, Mohamed Elkady, Molham Elbakary, Ahmed A Ibrahim, Magdy Eisa Saafan, Mohamed Osama Tomoum

**Affiliations:** 1 Otorhinolaryngology Department, Faculty of Medicine, Tanta University, El-Geish Street, Tanta, 31511 Egypt; 2 Ophthalmology Department, Faculty of Medicine, Tanta University, Tanta, Egypt; 3 Otorhinolaryngology Department, Faculty of Medicine, Alexandria University, Alexandria, Egypt

**Keywords:** Orbital, Transorbital, Clock model, Multiportal surgery, Skull base, Endoscopic endonasal approach, Superior eyelid approach, Lower eyelid preseptal approach

## Abstract

**Purpose:**

To present a road map and an algorithm that illustrate the rhinologist’s perspective in orbital surgery, guiding surgeons in selecting the most appropriate approach to orbital pathology.

**Methods:**

We conducted a prospective case series of one hundred consecutive patients undergoing orbital or transorbital endoscopic procedures from January 2023 to January 2025 at the Departments of Otorhinolaryngology and Ophthalmology, Tanta and Alexandria University Hospitals.

**Results:**

Using the right orbit as a clock, the endoscopic endonasal approaches provide access to the medial orbital compartment and orbital apex from 1 to 7 o'clock; the precaruncular approach allows access to the anterior medial compartment from 1 to 6 o'clock. The lower eyelid preseptal approach gives access to the inferior orbital compartment from 4 to 8 o'clock. The lateral retrocanthal or microorbitotomy approach offers access to the lateral orbital compartment from 7 to 11 o'clock. The Superior eyelid crease approach provides access to the superior compartment from 9 to 3 o'clock.

**Conclusion:**

The rhinologist’s perspective in orbital surgery has expanded from solely performing medial orbital and optic nerve decompression to bypass the periorbita to medial intraconal lesions and 360-degree access to the orbit by adding transorbital approaches.

**Supplementary Information:**

The online version contains supplementary material available at 10.1007/s00405-026-10155-6.

## Introduction

The orbit is an overlapping medical area, so a systematic workup including a complete ophthalmological examination, a CT scan, and/or an MRI, and a team-based assessment is recommended, involving an ophthalmologist, an otolaryngologist, and potentially a neurosurgeon if a craniotomy approach is being considered [1].

More than 100 different pathological conditions may affect the orbit, encompassing benign and malignant entities. These conditions are associated with variable manifestations, and the most common of which are proptosis, diplopia, and visual impairment [2]. Orbital pathologies can be traumatic, inflammatory, cystic, or neoplastic, and are classified into intraconal and extraconal lesions according to their location within the orbit relative to the extraocular muscle cone [3]. Previous authors have proposed ‘Round-the-Clock’ or 360° orbital mapping systems (Paluzzi et al., 2014; Castelnuovo et al., 2015; Locatelli et al., 2024). Our ‘Orbital Clock Concept’ builds on these foundations by applying ENT-based endoscopic anatomy to a 100-patient clinical series, emphasizing the integration of transnasal and transorbital corridors [4–6].

Meticulous preoperative planning is essential to safely address different orbital lesions while minimizing risks and promoting satisfactory aesthetic and functional outcomes [7]. A variety of open and, more recently, endoscopic endonasal and endoscopic-assisted (transorbital) approaches have been described to expose the orbit. Surgery has to be planned with diagnostic, curative, or decompressive intent based on the patient’s features, nature of the disease, and its radiological appearance. As the concept of minimally invasive endoscopic skull base surgery has markedly evolved in the last decades, the approach should create a portal to access lesions involving the orbit, sinuses, skull base, and intracranial structures while ensuring a good exposure of the anatomical structures with a minimally disruptive technique aiming to increase surgical safety and efficacy [6].

This study provides a road map and an algorithm to illustrate the rhinologist’s perspective in orbital surgery. It guides surgeons in choosing the most suitable approach for orbital pathology based on its location within the orbit relative to the optic nerve. Visualizing the orbit as a clock to simplify orientation and surgical planning aims to reduce the risk of adverse events and improve both aesthetic and functional outcomes.

## Patients and methods

This study was designed as a prospective descriptive case series involving one hundred consecutive patients undergoing orbital or transorbital endoscopic procedures over two years (January 2023 to January 2025) at the Departments of Otorhinolaryngology and Ophthalmology, Tanta and Alexandria University Hospitals. Patients with non-malignant pathologies affecting the orbit were included, while patients with malignant orbital tumors and unfit for surgery (severe bleeding tendency, coagulation disorder, or contraindication to general anesthesia) were excluded.

All patients were subjected to detailed history taking, complete general examination, clinical complete ophthalmological and otolaryngological examination, CT and MRI assessment of the lesion, and routine preoperative investigations. Preoperative multidisciplinary planning was done between the otolaryngologist and ophthalmologists to choose the most appropriate approach.

A key principle when choosing a surgical corridor is to avoid crossing the optic nerve to prevent direct manipulation and subsequent potential deficits. The right orbit was visualized as a clock (Fig. 5A), and the correlation between this model, the patients’ preoperative coronal CT scan and MRI images, and the approach with the exposure obtained was done.

We believed that different approaches could overlap in some areas when addressing the orbit. Therefore, primary and secondary outcome measures were addressed. The primary outcome was to assess the feasibility and anatomical applicability of the Orbital Clock framework. The secondary outcomes were surgical approach distribution, intraoperative complications, and postoperative visual or cosmetic results. 

### Statistical Analysis

All data were tabulated and analyzed using the statistical package for the social sciences software IBM SPSS Statistics for Windows, version 27 (IBM Corp., Armonk, N.Y., USA). Categorical data were presented as frequencies and percentages. Continuous data were tested for normality with the Shapiro–Wilk test. Normally distributed data were reported as mean ± standard deviation (SD), while skewed data were shown as the median and interquartile range (IQR) (25th–75th percentiles). Because this study was descriptive, only basic descriptive statistics (mean, range, frequency) were used; no hypothesis testing was performed.

## Results

This prospective case series study included 100 patients (45 males, 55 females) with various lesions affecting the orbit during the study period who underwent endoscopic endonasal, transorbital, or combined approaches. The patients’ age ranged from 6 to 67 years at the time of surgery (mean ± SD, 31.9 ± 19.7). At presentation, the clinical manifestations included proptosis (72%), limited mobility (68%), facial pain and headache (52%), eye swelling (40%), decreased visual acuity (17%), inferior dystopia (15%), relative afferent pupillary defect (RAPD) (14%), epiphora (7%), and diplopia (7%). The time elapsed between complaint and intervention ranged from 1 to 1800 days, with a median (IQR): 60.0 (14.0-189.0) days. Patients had various surgical indications including: subperiosteal abscess (18 patients), sinonasal mucoceles with orbital extension (16 patients), fungal sinusitis (10 patients), fibroosseous lesions (14 patients), tumors (16 patients), orbital wall fractures (15 patients), dacryocystoceles (5 patients and one of which was bilateral), thyroid orbitopathy (1 patient), and dermoid cysts (2 patients), and combined pathologies (3 patients with frontal osteoma and secondary frontal mucocele).

Regarding our primary outcomes, after reviewing all cases and correlating the radiological images with the approach used, the exposure obtained, and the clock model, we concluded that endoscopic endonasal approaches provide access to the medial orbital compartment and the orbital apex from 1 to 7 o’clock. The precaruncular approach offers access to the anterior medial compartment from 1 to 6 o’clock. The lower eyelid preseptal approach provides access to the inferior orbital compartment from 4 to 8 o’clock. The lateral retrocanthal or microorbitotomy approach provides access to the lateral orbital compartment from 7 to 11 o’clock. The superior eyelid crease approach provides access to the superior compartment from 9 to 3 o’clock. Both the superior and lateral orbital compartments can be accessed through the superior eyelid crease by extending its lateral limit, known as the access to the superolateral portal of the orbit from 7 to 3 o’clock (Fig. 5B).

For the secondary outcome, 91% of patients were managed with a single approach. Endoscopic endonasal access was achieved in 73 patients (73%), transorbital approaches were performed in 18 patients (18%), and combined (biportal) approaches were used in 9 patients (9%). Among transorbital approaches, the superior eyelid crease approach was most frequently used (11%), followed by lower eyelid preseptal (5%) and precaruncular (2%). Combined approaches included endoscopic endonasal with superior eyelid crease (5%), endoscopic with precaruncular (2%), endoscopic with lower eyelid preseptal (1%), and precaruncular combined with lateral orbitotomy (1%). Three patients were initially planned for an endonasal endoscopic approach preoperatively, but the decision was changed to a combined approach, as shown in Table 1.

**Table 1:** Clinical characteristics of the patients (*n* = 100)

Clinical characteristics							Value	
							Number	Percentage
Sex	Male						45/100	45%
	Female						55/100	55%
Age (years, Mean ± SD)							31.9 ± 19.7	
Etiology and approach used in each case	Medial Subperiosteal abscess					Endoscopic endonasal drainage	18/100	18%
	Mucoceles	Frontal				Endoscopic endonasal drainage	4/100	4%
		Ethmoid					11/100	11%
		Onodi cell					1/100	1%
	Tumors	Sphenoid wing meningioma				Superior eyelid crease incision with lateral extension (supplementary Fig. 1)	8/100	8%
		Orbital apex schwannoma				Endoscopic endonasal approach to orbital apex (supplementary Fig. 2) )	4/100	4%
		Orbital intraconal cavernous hemangioma				Endoscopic endonasal approach to the medial intraconal space (Fig. 1)	2/100	2%
		Recurrent frontal meningioma				Combined endoscopic endonasal and superior eyelid crease incision	2/100	2%
	Fungal sinusitis	Allergic fungal sinusitis				Endoscopic sinus surgery	7/100	7%
		Invasive fungal sinusitis causing orbital apex syndrome				Endoscopic endonasal debridement	3/100	3%
	Orbital Wall Fractures	Medial orbital wall fracture				Repair was done endoscopically endonasal in 2 patients, precaruncular in 2 patients, and through combined precaruncular and endonasal in the remaining 2 patients	6/100	6%
		Inferior orbital wall fractures				5 underwent lower eyelid preseptal repair (Fig. 2), while 2 patients underwent endoscopic endonasal repair, and the remaining one underwent a combined endoscopic and lower eyelid preseptal approach	8/100	8%
		Tension pneumorbita				Endoscopic medial orbital decompression	1/100	1%
	Dacryocystoceles					Endoscopic dacryocystorhinostomy	5/100	5%
	Thyroid orbitopathy					Combined precaruncular and lateral orbitotomy approaches (Fig. 3)	1/100	1%
	Fibroosseous lesions			Ossifying fibroma		Endoscopic endonasal resection	4/100	4%
				Fibrous dysplasia		Endoscopic endonasal resection	5/100	5%
				Osteoma		3 patients underwent a superior eyelid crease approach (Fig. 4), while 2 patients underwent a combined endoscopic endonasal and superior eyelid crease excision	5/100	5%
	Orbital dermoid cyst					Endoscopic endonasal approach to the medial intraconal space	2/100	2%
	Combined pathologies	CRSwNP with frontoethmoid osteoma and frontal mucocele				Endoscopic sinus surgery	2/100	2%
		Frontal osteoma and mucocele				Endoscopic endonasal and superior eyelid crease approach	1/100	1%
Approaches	Single approach (*N* = 91)		Endoscopic endonasal approach (EEA)				73/100	73%
			Transorbital (*N* = 18)		Superior eyelid crease (SLC)		11/100	11%
					Precaruncular (PC)		2/100	2%
					Lower eyelid preseptal (PS)		5/100	5%
	Combined (Biportal) (*N* = 9)				EEA + SLC		5/100	5%
					EEA and PC		2/100	2%
					EEA and PS		1/100	1%
					PC + lateral orbitotomy through canthotomy and cantholysis		1/100	1%

*N* number, *SD* standard deviation, *CRSwNP* chronic rhinosinusitis with nasal polyps, *EEA* endoscopic endonasal approach, *SLC* superior eyelid approach, *PC* precaruncular approach, *PS* lower eyelid preseptal approach

**Fig. 1:**
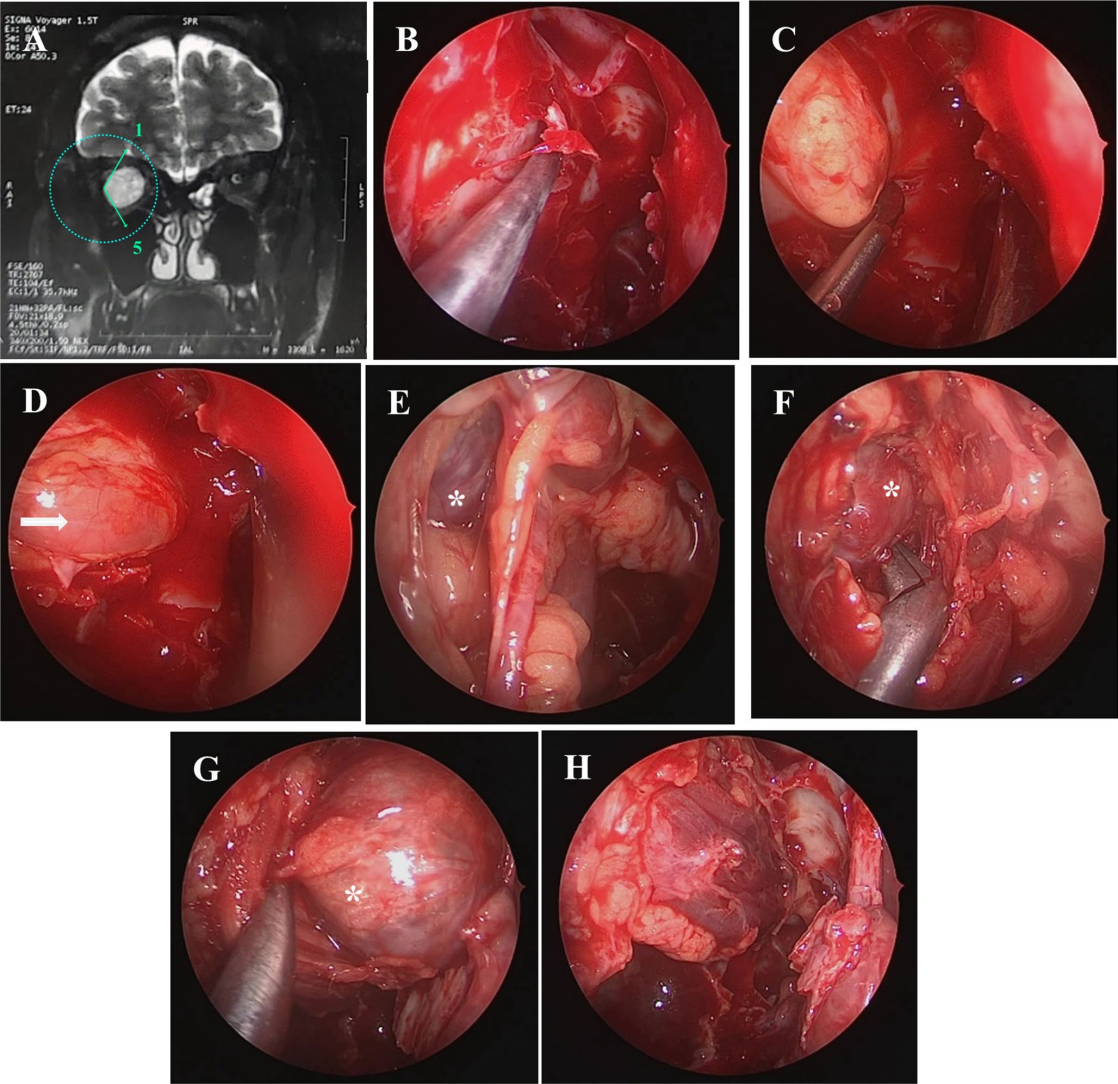
This 23-year-old man developed right-sided progressive proptosis caused by a medial intraconal lesion, primarily a cavernous hemangioma. The endoscopic endonasal approach was identified as the most direct and safest route to access this lesion. The hemangioma was completely removed, resulting in the complete resolution of proptosis without complications. **A** MRI coronal scan with overlying clock model showing the medial intraconal cavernous hemangioma between 1 and 5 o’clock, highlighting the extent of the orbit accessible through this approach. **B** Removal of lamina papyracea. **C** Incision of the periorbita using a sickle knife with herniation of the orbital fat. **D** Identification of the medial rectus muscle (white arrow). **E **and **F** Exposure of the intraconal space by retracting the medial rectus muscle, revealing the lesion (white asterisk). **G** Blunt dissection and removal of the intraconal lesion. **H** No reconstruction of the medial orbital wall was performed

**Fig. 2:**
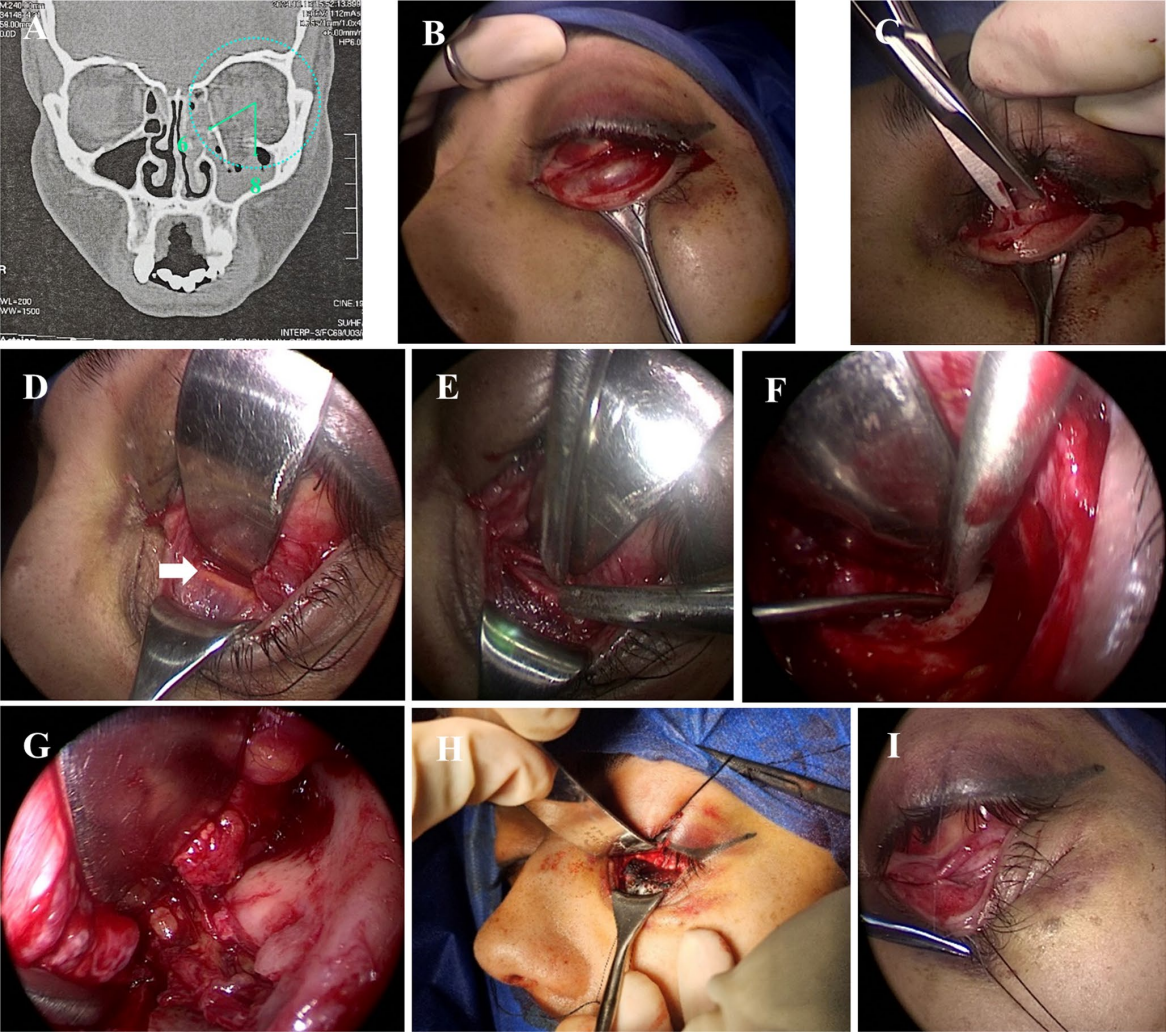
Surgical steps of lower eyelid presetal approach for the inferior orbital portal in case of orbital floor fracture with entrapment of the inferior rectus muscle. **A**) Coronal CT showing inferior orbital wall fracture with entrapped inferior rectus muscle and fat between 6-8 o’clock. **B**) Conjunctival incision for lower eyelid. **C**) Preseptal dissection to avoid orbital fat prolapse. **D**) Inferior orbital rim was encountered (white arrow). **E **and **F**) Subperiosteal dissection was done. **G**) Release of entrapped fat and the inferior rectus muscle. **H**) Reconstruction of the orbital floor using titanium mesh. **I**) Closure of conjunctival incision

**Fig. 3:**
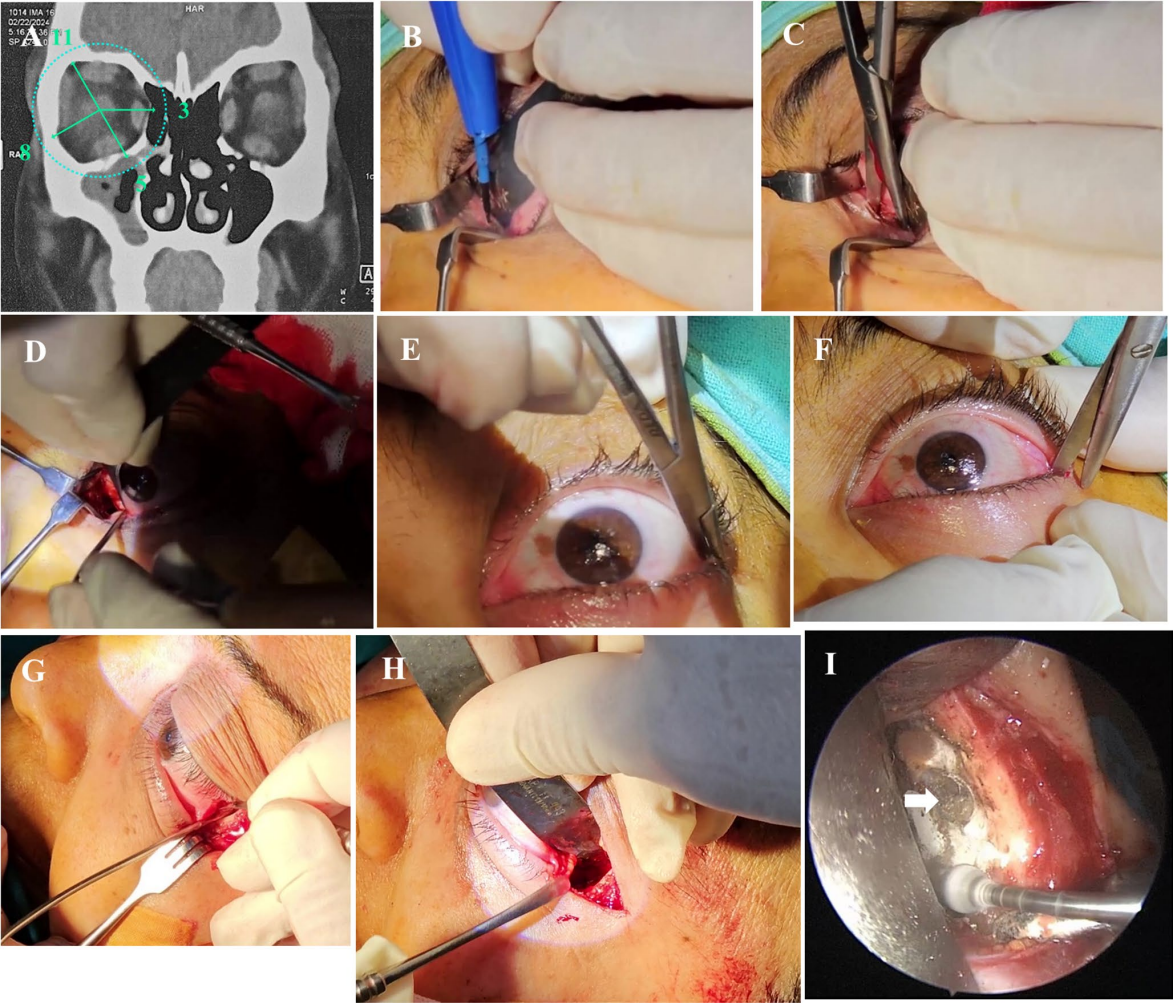
A 43-year-old woman developed exophthalmos and retro-orbital pain due to thyroid orbitopathy. Due to maxillary sinus infection and after interdisciplinary discussion, the decision was to use a precaruncular approach for medial orbital decompression between 3-5 o’clock, along with lateral canthotomy and cantholysis, and drilling of the zygomatic bone for lateral orbital decompression between 8-11 o’clock to keep the sterile orbit away from the contaminated nose. **A**) Coronal CT scan showing bilateral hypertrophied four recti muscles. **B**) Conjunctival incision with Colorado needle while protecting lacrimal puncta using retractors. **C**) Dissection to reach the subperiosteal plane using scissors. **D**) Outward fracture of the lamina papyracea using the freer. **E **and **F**) Crushing of the lateral canthal tendon, then cutting it with scissors. **G **and **H**) Release of periosteum using the freer to access the lateral subperiosteal plane. **I**) Drilling of the zygomatic bone in the lateral orbital wall was done until reaching the temporalis laterally (white arrow) using a diamond drill

**Fig. 4:**
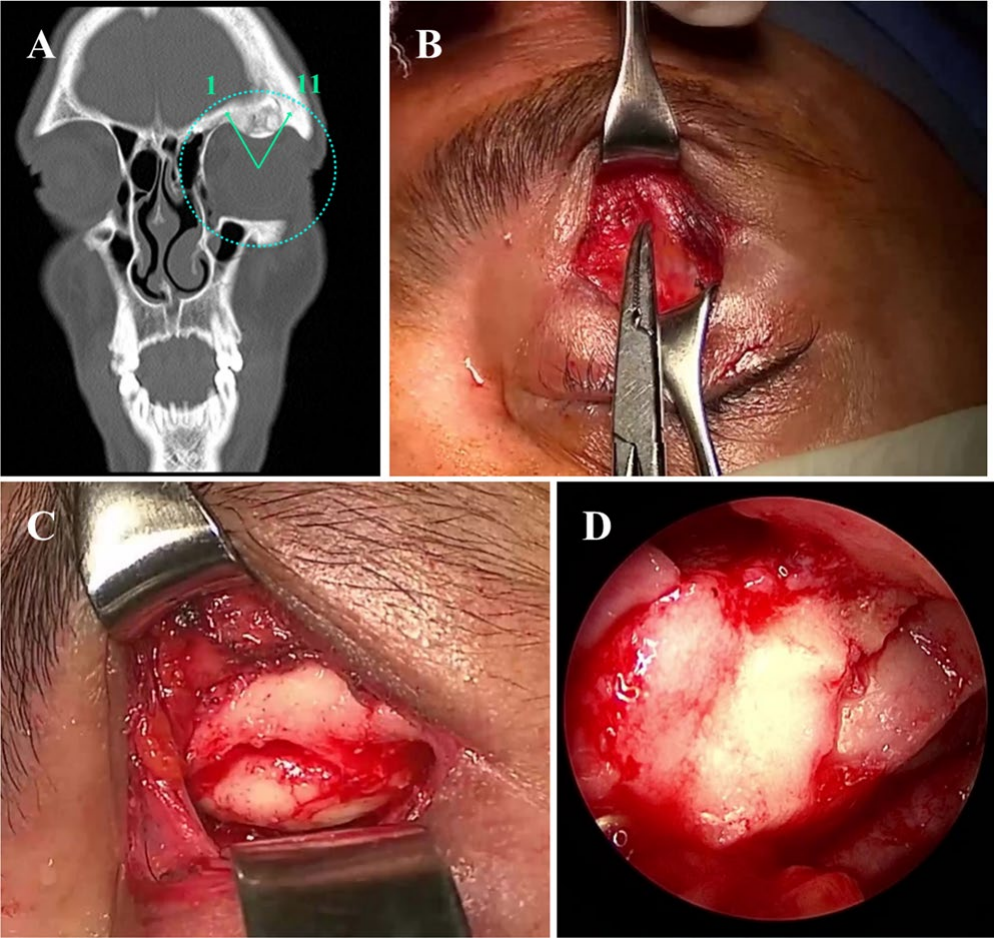
A 52-year-old man developed left inferior dystopia caused by a frontal osteoma pressing on his left eye. He had previous surgical interventions with unavailable previous operative or pathological details. The SLC was determined to be the most direct and safest route to access this lesion. The osteoma was completely excised, resulting in the full resolution of the dystopia without complications. **A**) Coronal CT scan showing the orbital osteoma between 1 and 11 o’clock after the model was flipped horizontally to match the left eye. **B**) A superior eyelid crease incision was made, followed by dissection through the orbicularis oculi. **D**) The superior orbital rim and osteoma were identified. **E**) Final view showing the site after removal of the osteoma

In one representative case, a 45-year-old male presented with a left frontoethmoid osteoma. Based on preoperative imaging and zonal localization within the proposed clock framework (1 o’clock to 4 o’clock), an endoscopic endonasal approach was initially planned. However, intraoperatively, the lesion demonstrated a broad-based attachment that limited safe and controlled resection through the endoscopic corridor alone. To ensure adequate exposure and complete removal, the surgical strategy was modified to a combined approach incorporating a superior eyelid crease incision. This adjunctive exposure provided direct superior access and improved visualization of the remaining part, allowing safe, controlled, and complete resection. This illustrates how approaches can integrate and overlap (Supplementary Fig. 3).

Intraoperatively, 98% of procedures were completed without complications. Cerebrospinal fluid (CSF) leak occurred in two patients (2%) during endonasal orbital and optic nerve decompression for extensive fibrous dysplasia and ossifying fibroma; both were repaired intraoperatively.

Postoperatively, 90% of patients experienced no complications. In the endoscopic endonasal group (*n* = 73), 91.7% had no postoperative complications. Reported events included nasal synechiae (4.1%), transient diplopia (1.4%), ecchymosis (1.4%), and one case of visual loss (1.4%) attributed to vascular injury during optic nerve decompression in extensive fibrous dysplasia. In the transorbital group (*n* = 18), 77.8% had no complications. Observed complications included ptosis (5.6%), enophthalmos with ptosis (5.6%), transient diplopia (5.6%), and one recurrence of hyperostosing sphenoid wing meningioma (5.6%). All patients in the combined (biportal) group (*n* = 9) had no postoperative complications (100%) as shown in Table 2.

**Table 2:** Frequency of intraoperative and postoperative complications

Complications			Number	Percentage
Intraoperative complications	No complications		98/100	98%
	CSF leak		2/100	2%
Postoperative complications	Endoscopic endonasal approaches (*n* = 73)	No Complications	67/73	91.7%
		Visual loss is primarily due to vascular injury of the optic nerve	1/73	1.4%
		Ecchymosis resolved after 10 days	1/73	1.4%
		Nasal synechiae managed by adhesiolysis	3/73	4.1%
		Diplopia resolved after 1 month	1/73	1.4%
	Transorbital (*n* = 18)	No Complications	14/18	77.8%
		Ptosis managed by an ophthalmologist	1/18	5.6%
		Enophthalmos and ptosis managed by an ophthalmologist	1/18	5.6%
		Diplopia resolved after 1 month	1/18	5.6%
		Recurrence of hyperostosing sphenoid wing meningioma	1/18	5.6%
	Combined (Biportal) (*n* = 9)	No complications	9/9	100%

## Discussion

Lesions involving the orbit have challenged surgeons for decades. Sometimes, the challenges are the lesions themselves, but in many other cases, major technical issues are related to the lesion’s location and the approach needed. The adoption of endoscopic technology in orbital surgery has been facilitated by technological advances, including angled endoscopes, high-definition monitors, navigation systems, high-resolution imaging, and improved anatomical knowledge. This has led to the development of minimally invasive techniques that can reduce overall surgical morbidity while achieving satisfactory aesthetic and visual outcomes [8].

The chosen approach to the orbital lesion varies based on its position, the relationship with near neurovascular structures, the suspected nature of the lesion, and the surgeon’s expertise [9].

Endoscopic and endoscopic-assisted approaches to orbital lesions have provided surgeons with alternatives to the previous traditional open approaches, such as craniotomies, resulting in less visual morbidity, avoiding disfiguring external scars, extensive bony work, long operative time, additional brain retraction, and prolonged recovery periods [10–11].

Since being introduced in 2010 by Kris Moe, numerous surgeons worldwide have performed transorbital neuroendoscopic surgery (TONES). With careful preoperative planning, proper patient selection, and surgical technique, TONES provides a highly effective, minimally invasive, and safe alternative to previously reported procedures when used alone or as a part of multiportal combination with transnasal or other approaches to operate within or through the orbit on the skull base, brain, and paranasal sinuses with no significant neurological or medical complications, just enophthalmos and ptosis, and no significant blood loss like our results [12, 13].

**Fig. 5:**
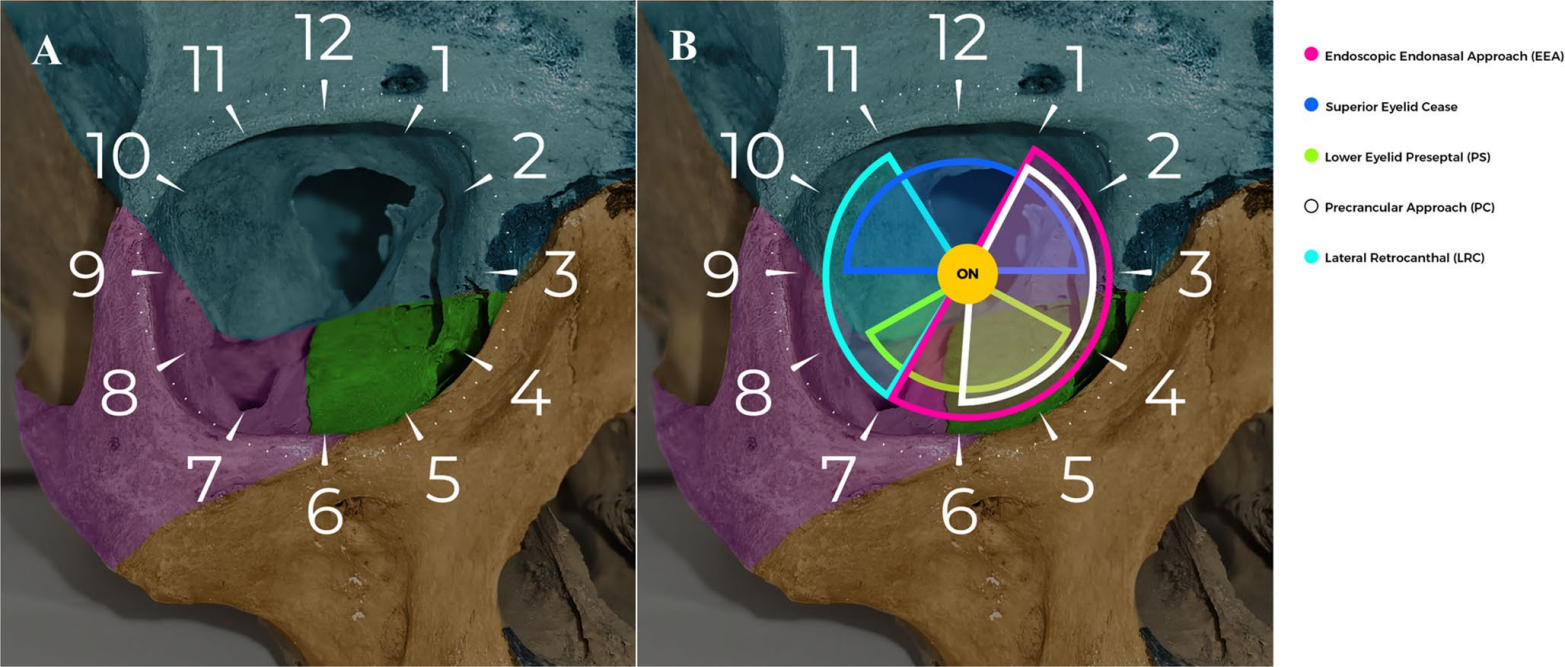
(5**A**): The right orbit of a dried skull was viewed as a clock. (5**B**) A diagrammatic clock model of the right orbit illustrating the integration and overlap of the various surgical approaches. ON, optic nerve. The color code is beside the figure

So, based on our case series of 100 cases, a model like a clock (Fig. 5B), similar to Paluzzi et al., [4] is proposed to illustrate the rhinologist’s perspective in orbital surgery. It will guide surgeons in choosing the most appropriate approach to orbital pathology according to the location of the pathology within the orbit relative to the optic nerve, based on using a minimally disruptive technique with better aesthetic outcomes and the goal of increasing surgical safety and efficacy.

The difference is that we conducted a multicenter prospective case series study including one hundred patients using endoscopically assisted transorbital surgery instead of traditional neurosurgical approaches to expose the orbit’s superior, medial, inferior, and lateral compartments, highlighting the role of the rhinologist in orbital surgery and emphasizing minimally invasive endoscopic corridors whenever feasible. We used the right orbit to demonstrate the model starting in a clockwise manner, which can be flipped horizontally to be applied to the left orbit. We found that endoscopic endonasal approaches provide access to the medial orbital compartment and orbital apex from 1 to 7 o’clock, the precaruncular approach provides access to the anterior medial compartment from 1 to 6 o’clock, and this is consistent with the Paluzzi model. However, the lower eyelid preseptal approach provides access to the inferior orbital compartment from 4 to 8 o’clock instead of using zygomatic osteotomy. When approaching lateral and superior orbital lesions, instead of using frontotemporal craniotomy, the lateral retrocanthal or lateral microorbitotomy approach provides access to the lateral orbital compartment with more exposure from 7 to 11 o’clock, unlike 8 to 10 o’clock in the Paluzzi model. Superior eyelid crease approach provides access to the superior compartment from 9 to 3 o’clock. Both superior and lateral orbital compartments can be accessed through the superior eyelid crease by extending its limit laterally (supplementary Fig. 1B), so-called access to the superolateral portal of the orbit from 7 to 3 o’clock. To ensure comprehensive access to the full intra- and extraconal orbit (360 degrees), these approaches should not be used independently but often need to be combined to provide the most suitable choice for the patient and a specific pathology, known as multiportal surgery.

The proposed “Orbital Clock” concept provides a structured anatomical framework to facilitate spatial orientation and guide surgical corridor selection, communication among surgeons, and approach planning after fulfilling several additional factors, including the underlying suspected pathology, the relationship to critical structures such as the optic nerve and extraocular muscles, the extent of the lesion, the presence of intracranial extension may necessitate combined or alternative approaches, patient-related considerations (prior surgeries, cosmetic concerns), and the surgeon expertise and institutional experience.

Orbital surgery requires knowledge of the detailed anatomy, clear understanding of the pathologies that may be encountered, adequate preoperative planning and patient counseling, knowledge of the expected morbidities, and multidisciplinary team evaluation to allow safe traverse, and also provide excellent access to the sino-orbito-cranial interface because of the close relationship between the orbital contents, the nose and paranasal sinuses, and the anterior and middle cranial fossae [5].

When a lesion lies within a zone technically accessible through multiple surgical corridors, the preferred strategy is typically the one that provides adequate surgical exposure while minimizing overall morbidity and unnecessary manipulation of critical neurovascular structures, and achieving good functional and aesthetic outcomes. Furthermore, surgeon familiarity and technical expertise with a given approach are pivotal for optimizing safety and efficiency.

## Conclusion

For full and effective orbital access, the skilled surgeon’s armamentarium should include a variety of surgical approaches to access the orbit from different angles. The rhinologist’s perspective in orbital surgery has expanded from just performing medial orbital and optic nerve decompression to bypass the periorbita to medial intraconal lesions and 360-degree access to the orbit by adding transorbital approaches. The orbit is not just a site of pathological events, but can be used as an efficient corridor to reach nearby and deeper areas when traversed safely.   

## Electronic Supplementary Material

Below is the link to the electronic supplementary material.

Supplementary Material 1. Surgical steps of superior eyelid crease incision with lateral extension for the superolateral portal used in treating hyperostosing sphenoid wing meningioma. (A) Coronal CT scan displaying hyperostosing meningioma from 8 to 12 o’clock. (B) Marking the superior eyelid crease incision with lateral extension. (C) Making the incision. (D) Dissecting through the orbicularis oculi muscle. (E) Identifying the superior orbital rim and initiating subperiosteal dissection. (F) A hyperostotic bony lesion (black oval line). (G) Drilling the lesion. (H) Exposure of the temporalis muscle laterally (white arrow) and approaching the dura posteriorly (asterisk).

Supplementary Material 2. Surgical steps of endoscopic endonasal approach for orbital apex lesions. (A) Coronal MRI imaging showing left orbital apex schwannoma between 2 and 5 O’clock. (B) Lesion appeared after complete maxillary antrostomy and sphenoethmoidectomy. C and D) Removal of the bone over the lesion. E) The core of the lesion was opened (blue arrow), and piecemeal biopsies were taken. F) Separation of the sac wall from the periorbita. G) Removal of sac. H) Lesion was removed, and the optic nerve appeared (white arrow).

Supplementary Material 3. Surgical steps of combined endoscopic endonasal and superior eyelid crease incision for left frontoethmoid osteoma. (A) Coronal CT imaging showing left frontoethmoid osteoma between 1 and 4 O’clock. (B) The osteoma appeared after removing the overlying mucosa (white arrow). (C) Attempt to gain a plane of cleavage between the osteoma and the lateral nasal wall. (D) Drilling of the osteoma. (E) The remaining part of the osteoma (blue arrow). (F) Superior eyelid crease incision. (G) Transorbital drilling of the remaining part of the osteoma. (H) Postoperative coronal CT imaging showing complete removal of the osteoma.

## Data Availability

The datasets used during the current study are available from the corresponding author on reasonable request.

## Declarations

### Type of Article

Original article.
