# Supplementary figures and images for: Orbital clock concept: A novel anatomical zoning system for endoscopic vs. external orbital approaches
Source: Eur Arch Otorhinolaryngol. 2026 Apr 29;283(6):3733–42. doi: 10.1007/s00405-026-10155-6 (PMC13249908; doi:10.1007/s00405-026-10155-6)

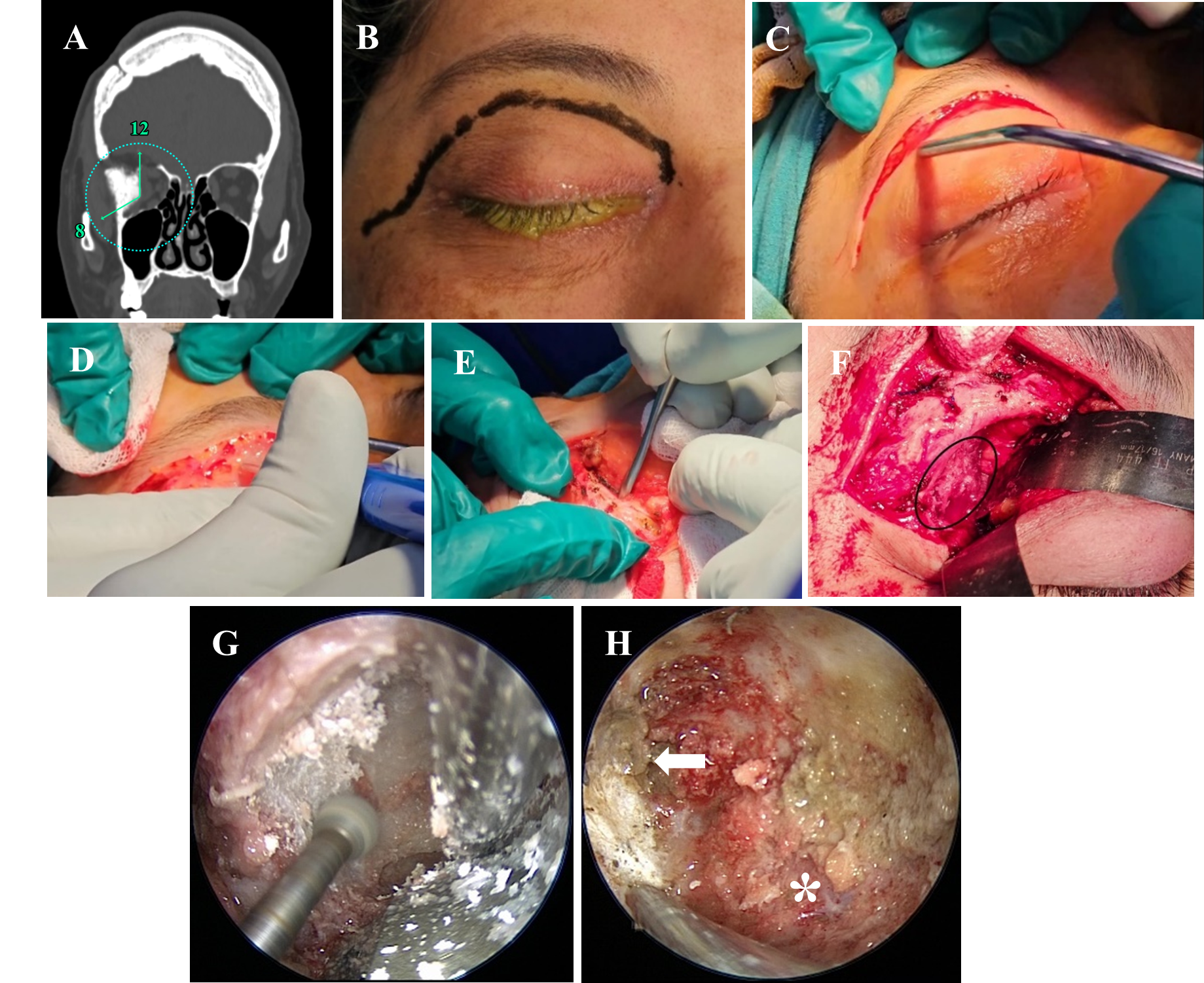

Supplement: Supplementary file 1 — Supplementary Material 1. Surgical steps of superior eyelid crease incision with lateral extension for the superolateral portal used in treating hyperostosing sphenoid wing meningioma. (A) Coronal CT scan displaying hyperostosing meningioma from 8 to 12 o’clock. (B) Marking the superior eyelid crease incision with lateral extension. (C) Making the incision. (D) Dissecting through the orbicularis oculi muscle. (E) Identifying the superior orbital rim and initiating subperiosteal dissection. (F) A hyperostotic bony lesion (black oval line). (G) Drilling the lesion. (H) Exposure of the temporalis muscle laterally (white arrow) and approaching the dura posteriorly (asterisk). [file 405_2026_10155_MOESM1_ESM.png]

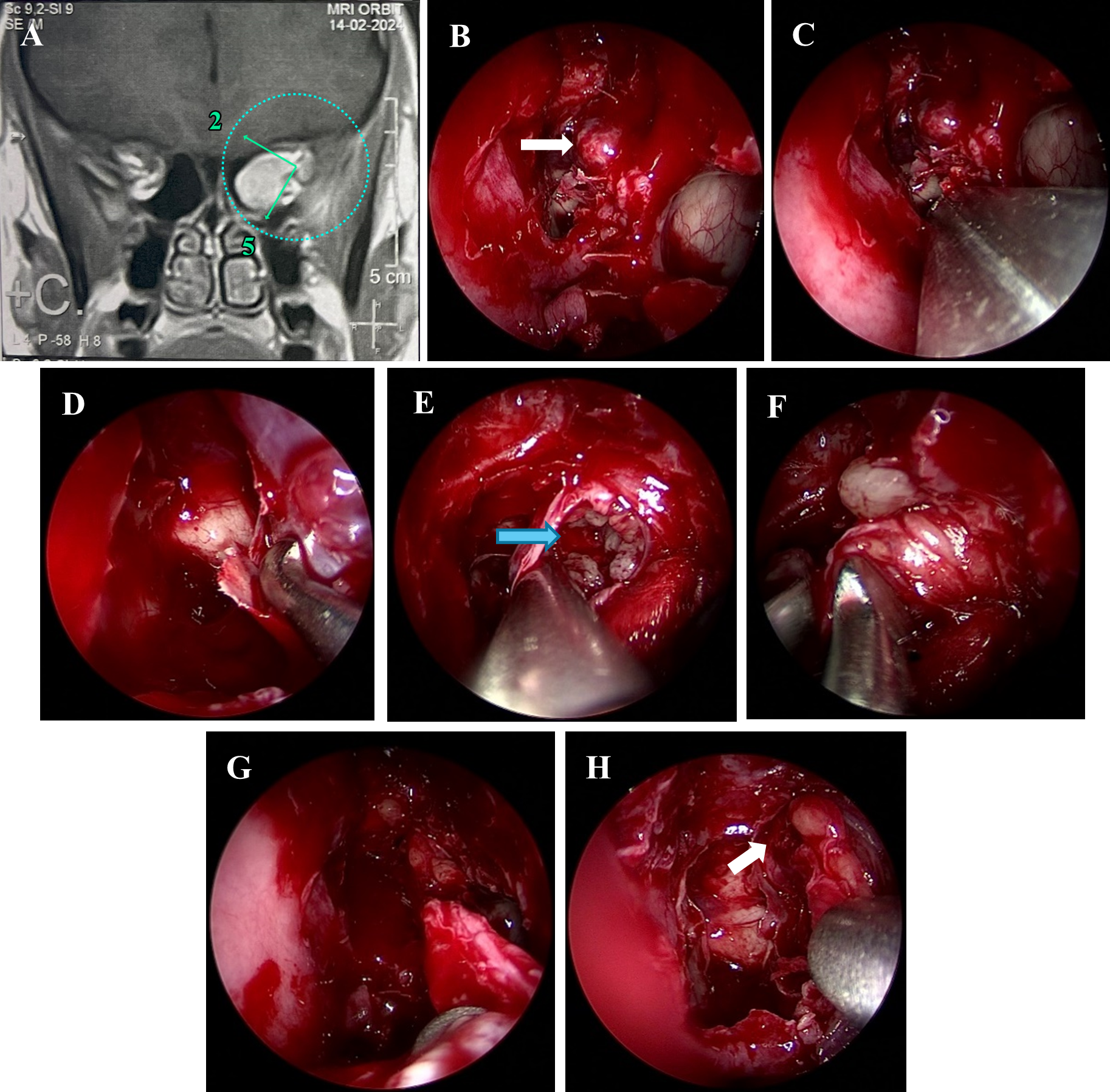

Supplement: Supplementary file 2 — Supplementary Material 2. Surgical steps of endoscopic endonasal approach for orbital apex lesions. (A) Coronal MRI imaging showing left orbital apex schwannoma between 2 and 5 O’clock. (B) Lesion appeared after complete maxillary antrostomy and sphenoethmoidectomy. C and D) Removal of the bone over the lesion. E) The core of the lesion was opened (blue arrow), and piecemeal biopsies were taken. F) Separation of the sac wall from the periorbita. G) Removal of sac. H) Lesion was removed, and the optic nerve appeared (white arrow). [file 405_2026_10155_MOESM2_ESM.png]

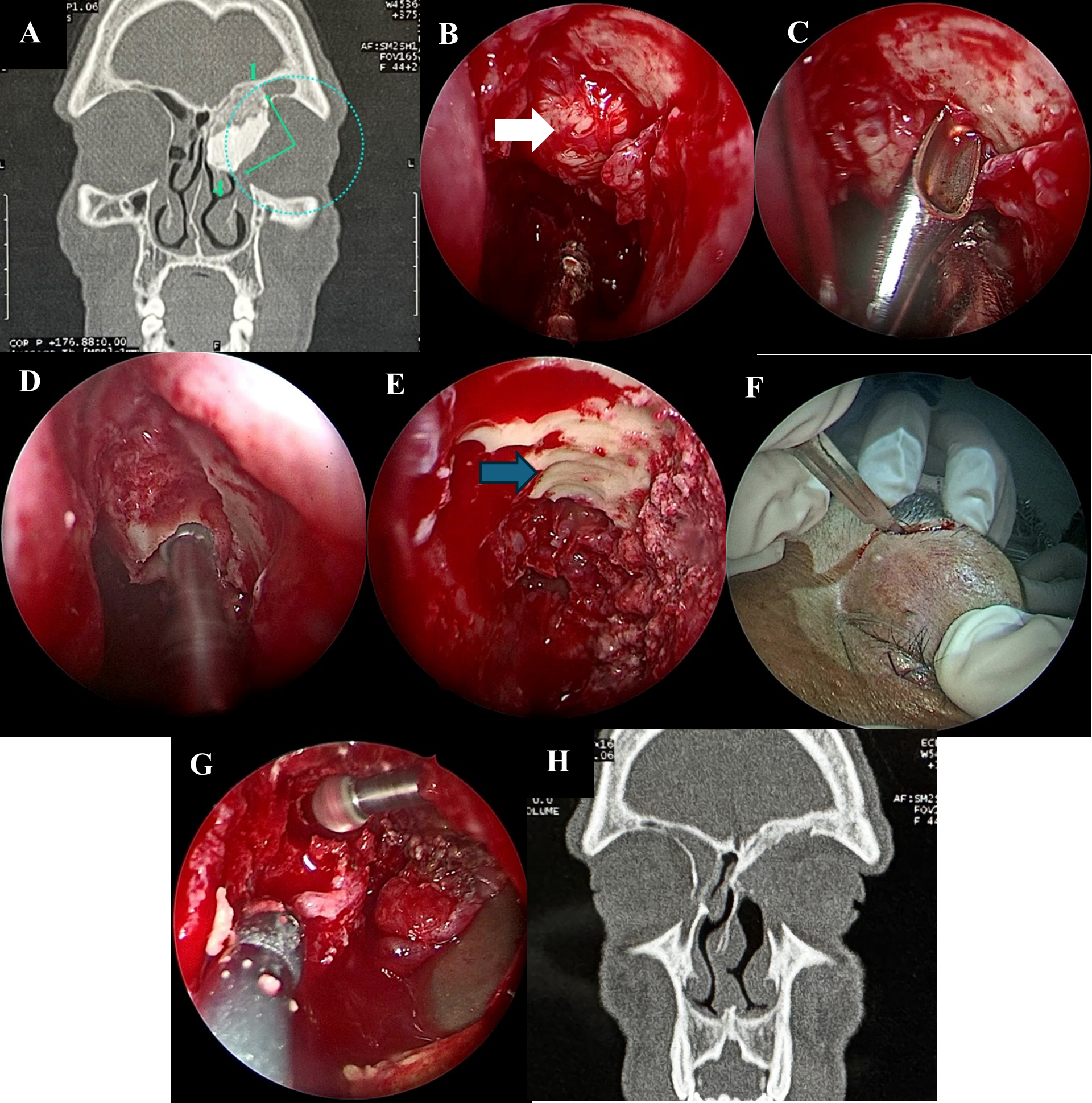

Supplement: Supplementary file 3 — Supplementary Material 3. Surgical steps of combined endoscopic endonasal and superior eyelid crease incision for left frontoethmoid osteoma. (A) Coronal CT imaging showing left frontoethmoid osteoma between 1 and 4 O’clock. (B) The osteoma appeared after removing the overlying mucosa (white arrow). (C) Attempt to gain a plane of cleavage between the osteoma and the lateral nasal wall. (D) Drilling of the osteoma. (E) The remaining part of the osteoma (blue arrow). (F) Superior eyelid crease incision. (G) Transorbital drilling of the remaining part of the osteoma. (H) Postoperative coronal CT imaging showing complete removal of the osteoma. [file 405_2026_10155_MOESM3_ESM.png]
